# Investigating Repeat Expansions in *NIPA1*, *NOP56*, and *NOTCH2NLC* Genes: A Closer Look at Amyotrophic Lateral Sclerosis Patients from Southern Italy

**DOI:** 10.3390/cells13080677

**Published:** 2024-04-14

**Authors:** Paola Ruffo, Francesca De Amicis, Vincenzo La Bella, Francesca Luisa Conforti

**Affiliations:** 1 Medical Genetics Laboratory, Department of Pharmacy, Health and Nutritional Sciences, University of Calabria, 87036 Rende, Italy; paola.ruffo@unical.it; 2 Neuromuscular Diseases Research Section, National Institute on Aging, Bethesda, MD 20892, USA; 3 Department of Pharmacy, Health and Nutritional Sciences, University of Calabria, 87036 Rende, Italy; francesca.deamicis@unical.it; 4 ALS Clinical Research Centre and Laboratory of Neurochemistry, Department of Experimental Biomedicine and Clinical Neurosciences, University of Palermo, 90133 Palermo, Italy; vincenzo.labella@unipa.it

**Keywords:** amyotrophic lateral sclerosis, NIPA1, NOP56, NOTCH2NLC, repeats expansion

## Abstract

The discovery of hexanucleotide repeats expansion (RE) in Chromosome 9 Open Reading frame 72 (*C9orf72)* as the major genetic cause of amyotrophic lateral sclerosis (ALS) and the association between intermediate repeats in Ataxin-2 (*ATXN2)* with the disorder suggest that repetitive sequences in the human genome play a significant role in ALS pathophysiology. Investigating the frequency of repeat expansions in ALS in different populations and ethnic groups is therefore of great importance. Based on these premises, this study aimed to define the frequency of REs in the *NIPA1*, *NOP56,* and *NOTCH2NLC* genes and the possible associations between phenotypes and the size of REs in the Italian population. Using repeat-primed-PCR and PCR-fragment analyses, we screened 302 El-Escorial-diagnosed ALS patients and compared the RE distribution to 167 age-, gender-, and ethnicity-matched healthy controls. While the REs distribution was similar between the ALS and control groups, a moderate association was observed between longer RE lengths and clinical features such as age at onset, gender, site of onset, and family history. In conclusion, this is the first study to screen ALS patients from southern Italy for REs in *NIPA1*, *NOP56*, and *NOTCH2NLC* genes, contributing to our understanding of ALS genetics. Our results highlighted that the extremely rare pathogenic REs in these genes do not allow an association with the disease.

## 1. Introduction

Amyotrophic lateral sclerosis (ALS), also known as “Lou Gehrig’s disease” or “Charcot’s disease” is a progressive neurodegenerative disease (ND) characterized by the degeneration of the motor neurons in the brain and spinal cord with consequent muscle weakness and eventual paralysis resulting in death within 2–5 years of symptom onset [1]. The onset can be bulbar or spinal. In the first case, the disease manifests itself with an initial weakness in the facial muscles, tongue, and pharynx, while in the spinal onset affected muscles are distal, in the upper or lower limbs [2].

Sporadic ALS (sALS) is the most common form of the disease, accounting for some 90% of all cases [3,4]. A family history of ALS is present in approximately 10% of cases with a genetic cause present in ~15% of patients showing an autosomal dominant pattern [5]. Over half of the familial cases are due to pathogenic mutations in disease-associated genes, among which *SOD1* (Superoxide Dismutase 1, OMIM * 147450), *FUS* (Fused in Sarcoma, OMIM * 137070, *TARDBP* (Tar DNA-Binding Protein, OMIM * 605078), and *C9orf72* (Chromosome 9 Open Reading Frame 72, OMIM * 614260) are the most frequently involved [3,6]. The hexanucleotide (GGGGCC) repeat expansion (RE) in the non-coding region of the *C9orf72* gene is the most frequent genetic cause in ALS patients [7,8].

Over the last decade, numerous studies have focused on discovering the role of repeat expansions (REs) other than *C9orf72* in ALS, such as *ATXN1* (Ataxin-1, OMIM * 601556) and *ATXN2* (Ataxin-2, OMIM * 601517). Repeat expansions in these genes have been primarily associated with other NDs, such as spinocerebellar ataxias (SCA) and hereditary spastic paraplegia (HSP) [9,10]. Furthermore, copy number variations (CNVs) in ALS have recently been the research focus of [11]. Excluding REs in *C9orf72*, 17.6% of individuals who were clinically diagnosed with ALS or frontotemporal dementia (FTD) had at least one short expansion tandem repeat (STR) allele [12] reported as pathogenic or intermediate for other NDs such as *ATXN1* (spinal cerebellar ataxia type, SCA1), *ATXN2* (SCA2), *ATXN8* (Ataxin 8, OMIM *613289, SCA8), *TBP* (TATA box-binding protein OMIM *600075, SCA17), *HTT* (Huntingtin, OMIM * 613004, Huntington’s disease), *DMPK* (Dystrophia Myotonica Protein Kinase OMIM * 160900, Myotonic Dystrophy 1, DM1), *CNBP* (Cchc-Type Zinc Finger Nucleic Acid-Binding Protein OMIM * 116955, DM2), and *FMR1* (Fragile X Messenger Ribonucleoprotein 1 OMIM * 309550, fragile X disorders) [12,13,14].

Based on this scientific evidence, several studies have been conducted evaluating the role of REs in other ALS-related genes. Among them, increased interest is aimed at the *NIPA1* gene (non-imprinted gene in Prader–Willi/Angelman syndrome region protein 1, OMIM * 608145). Mutations in this gene are known to cause HSP type 6, a progressive ND with slow upper motor neuron signs. Blauw and collaborators found an association between rare deletions in *NIPA1* and ALS by applying a whole-genome screening [15]. The same group, in 2012, showed that long or expanded polyalanine repeats in this gene confer greater disease susceptibility [16]. *NIPA1* is increasingly recognized as a genetic risk factor in ALS, with rare deletions and expanded polyalanine repeats linked to elevated disease risk. These variants may worsen motor neuron degeneration and influence disease severity. *NIPA1’s* involvement in cellular trafficking underscores its potential role in ALS pathology, offering avenues for targeted therapies and personalized treatment strategies. Further research into *NIPA1*-associated ALS is crucial for advancing our understanding of the disease and developing effective interventions [15].

Another hexanucleotide repeat expansion potentially associated with ALS is the *NOP56* (Nucleolar Protein 56) gene. The long REs of this gene cause SCA36, a group of autosomal dominant NDs in which prominent motor neuron degeneration is present [17,18]. Several studies revealed a motor neuron involvement including weak expression of *NOP56*, *TARDBP*, and *FUS* in an ALS mouse model, which happens before the seeming onset of the disease [19]. Nonetheless, few studies have been led currently on the association between ALS and the REs size within *NOP56*. While *NOP56* mutations are relatively rare compared to other genetic contributors to ALS, studies have implicated *NOP56* in the molecular pathways underlying motor neuron degeneration. Although the exact mechanisms by which *NOP56* mutations contribute to ALS pathology remain to be fully elucidated, their association with SCA suggests potential involvement in motor neuron dysfunction and degeneration [20].

Otherwise, other studies have already analyzed the probable correlation between REs in the *NOTCH2NLC* (Notch2 N-Terminal-Like C, OMIM * 618025) gene and ALS [21,22]. In particular, trinucleotide expansions of this gene have been classified as pathogenetic for NIID (neuronal intranuclear inclusion disease), which shows clinical overlaps with ALS [1,23,24]. The precise mechanisms by which *NOTCH2NLC* expansions contribute to ALS pathophysiology remain to be fully elucidated, but their identification underscores the genetic heterogeneity of ALS and highlights this gene as a novel candidate gene in ALS research. Understanding the clinical relevance of *NOTCH2NLC* in ALS may provide valuable insights into disease mechanisms and could ultimately lead to the development of targeted therapies for ALS patients with expansions in this gene [25].

In this study, we aimed to (i) define the frequency of REs in *NIPA1*, *NOP56,* and *NOTCH2NLC* in a large cohort of Italian ALS cases; and (ii) discover the potential associations between phenotypes and the size of REs within *NIPA1*, *NOP56,* and *NOTCH2NLC* genes.

## 2. Materials and Methods

### 2.1. Population

The study cohort included 302 patients (54% male, 46% female) with ALS from southern Italy who were prospectively recruited and randomized. All patients underwent a comprehensive neurological evaluation to establish the clinical diagnosis of ALS according to the Gold Coast Criteria [26] which represent the evolution of the revised El-Escorial criteria [27]. The demographic and clinical characteristics of the South Italian ALS cohort are described in Table 1. All individuals provided written informed consent for participation in genetic studies for research purposes. The control cohort (CTRL) included 167 Italian individuals, comprising both healthy samples (*n* = 31) and individuals diagnosed with other clinical conditions (*n* = 136) matched by gender, age, and geographic region. All samples (ALS and CTRL) were analyzed using the same pipeline.

### 2.2. Genetic Analysis

Genomic DNA was extracted from peripheral blood using a standard extraction method. Each patient underwent pathogenic mutations in genetic screening for the most common pathogenic ALS-related genes, i.e., *SOD1*, *TARDBP*, *FUS,* and *C9orf72* [28].

**Figure 1:**
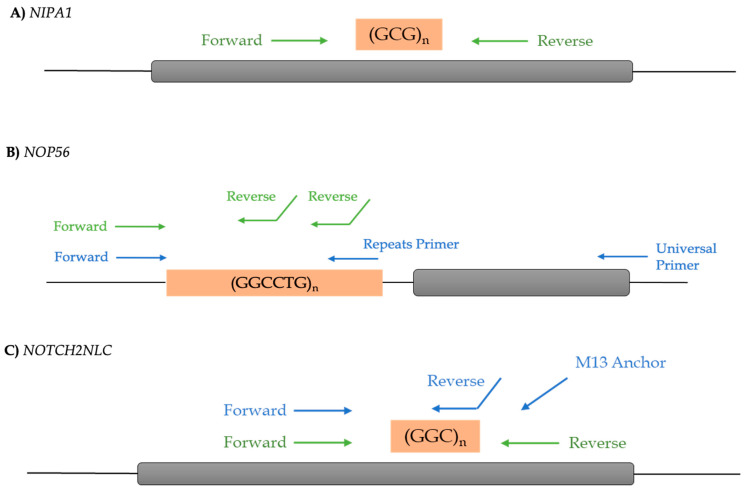
Oligonucleotides design in *NIPA1*, *NOP56,* and *NOTCH2NLC* genes to REs study. (**A**) In the fluorescence amplicon length analysis (green), the fluorescent-labelled primers flank the region comprising the *NIPA1* repeat (GCG). (**B**) Schematic representation of the *NOP56* gene. In the fluorescence amplicon length analysis (green), the reverse primer binds at multiple sites within GGCCTG repeat alleles, giving rise to a mixture of products. In the repeat-primed PCR (blue), the universal primer preferentially binds to the end of products from previous amplification rounds owing to the stabilizing effect of the 5′ tail sequence. (**C**) Schematic representation of the *NOTCH2NLC* gene. The disease associated RE (orange box) was identified in exon 1 of the NM_001364012 isoform. Green and blue arrows show the primers using fluorescence amplicon length analysis and repeat-primed PCR, respectively. (Created with BioRender).

The REs in *NIPA1*, *NOP56,* and *NOTCH2NLC* were screened by both repeat-primed PCR (polymerase chain reaction) and PCR-fragment analyses using primer pairs flanking the repeat. Considering that sample quality is critical in several genomic experiments, 295 DNA samples underwent genetic RE analysis of which 269 were from sporadic ALS cases and 26 with family history. In particular, genotyping of the GCG repeat in the first exon of *NIPA1* was performed by fragment-length analysis with fluorescent primers, as already described [16] (Figure 1, Supplementary Figure S1).

To detect the hexanucleotide repeat (GGCCTG) in the first intron of the pre-mRNA processing gene *NOP56*, a conventional PCR method was applied with a pair of fluorescently labelled primers. Homozygous samples with normal repeats discovered by conventional PCR were examined with repeat-primed PCR (sequence of primers used according to [29] (Figure 1, Supplementary Figure S1)).

For the genotyping of the GGC repeat in the first *NOTCH2NLC* exon, we conducted a fluorescent amplicon length analysis and, subsequently, a repeat-primed PCR to determine the right number of REs, as described [23] (Figure 1, Supplementary Figure S1). The product fragments were sized by capillary electrophoresis on a SeqStudio Genetic Analyzer (Thermo Fisher Scientific, Waltham, MA, USA) with GeneScan 500 ROX dye Size Standard (Thermo Fisher Scientific, Waltham, MA, USA). The data analysis was performed by the GeneMapper 6 program and the length of the highest signal peak of the expanded allele was used to estimate the RE. The sizes of the alleles were used to calculate the repeat number according to the reference human genome hg19. The repeat size has been confirmed by Sanger sequence analysis. All the primer sequences are displayed in Supplementary Table S1.

### 2.3. Statistical Analysis

To measure the binding between polynucleotide REs and several clinical features of ALS patients, we employed the chi-square test (χ^2^), Fisher’s exact test (for categorical variables), Student’s *t*-test (for continuous variables), and the odds ratio (OR) method. In particular, we performed these analyses on all subgroups of ALS and control samples, correlating the REs length in *NIPA1*, *NOP56,* and *NOTCH2NLC* with age at onset, gender, site of onset, family history, Amyotrophic Lateral Sclerosis Functional Rating Scale-Revised (ALSFRS-R) score, and disease progression rate (ΔFS). ΔFS is calculated as in [30].

(1) $$\Delta FS=\frac{\left[48-\left(Total\;ALSFRS-R\;score\;at\;initial\;assessment\right)\right]}{Time\;from\;onset\;of\;symptoms\;to\;initial\;assessment\;(months)}$$

Specifically, we dichotomize the ΔFS values into <0.7 and ≥0.7, with 0.7 being a median ΔFS value in large populations [31] Previous studies have shown that ALS patients with a ΔFS ≥ 0.7 tend to have shorter survival times and more aggressive disease courses than those with slower progression rates [32]

Kaplan–Meier univariate analysis was used to determine the effect of polynucleotide REs on survival time. All statistical analyses were performed using GraphPad Prism v.7. Differences with *p*-value < 0.05 were considered statistically significant.

To investigate the association between REs length and survival, we conducted a Kaplan–Meier analysis using the statistical software R (R 4.3.). A Winsorized test was applied to manage the potential influence of extreme survival values or outliers in the data set [33]. The survival curves were generated to visualize survival probabilities over time for the normal and long REs subgroups. The log-rank test was used to assess the significance of differences in survival distributions between these subgroups, and the resulting log-rank *p*-value was used to measure statistical significance.

## 3. Results

### 3.1. NIPA1

**Figure 2:**
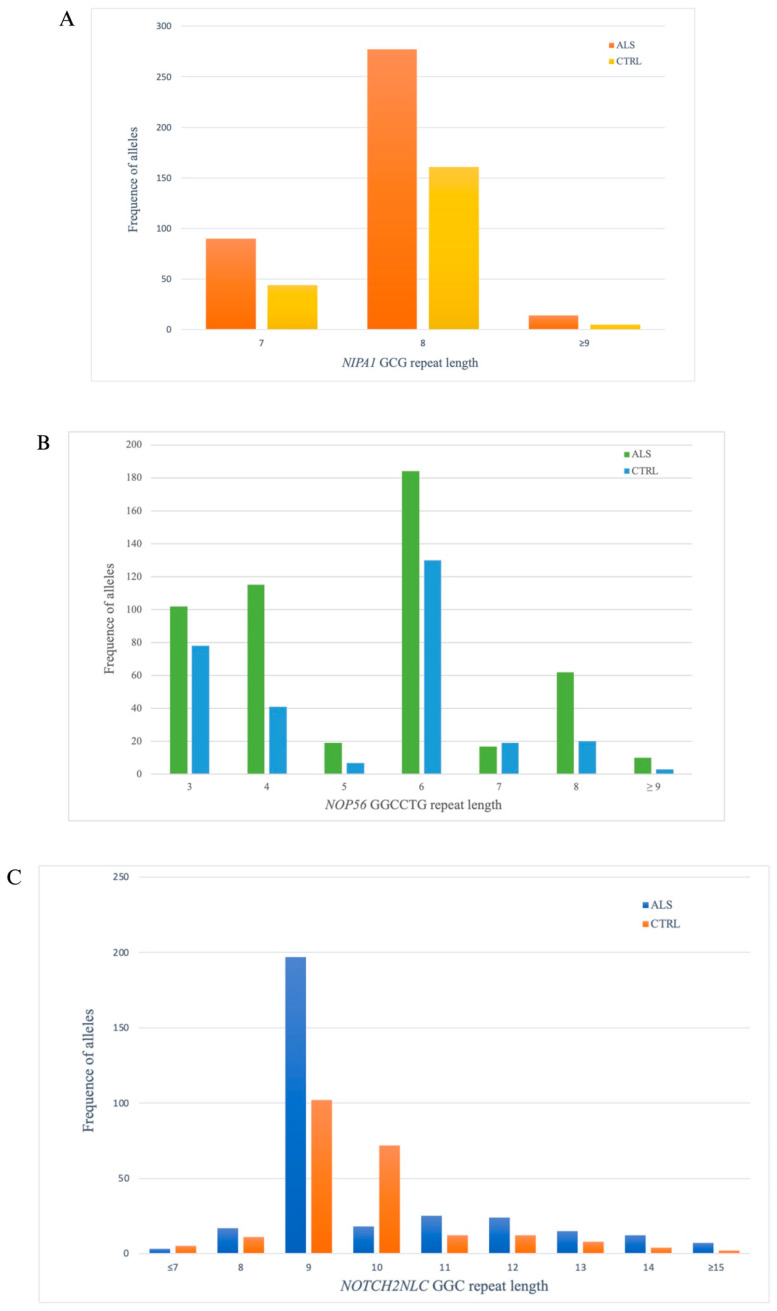
Distribution of allele frequency and allele size in ALS patients and controls. REs length in the alleles of (**A**) *NIPA1*, (**B**) *NOP56,* and (**C**) *NOTCH2NLC*.

Exon 1 of *NIPA1* encodes a GCG repeat which most often results in an allele length of 7 or 8 in healthy controls. The *NIPA1*-REs ranged from 7 to 10 repeats (GCG_7_–GCG_10_ alleles) in ALS patients and from 7 to 8 repeats (GCG_7_–GCG_8_ alleles) in control samples. The RE distribution between ALS and control cases was similar (*p* = 0.39) with the most frequent alleles being GCG_7_ (17.1%) and GCG_8_ (80.7%) (Figure 2A).

In order to evaluate the effect of the different REs lengths, we split the *NIPA1* alleles into “short” (GCG_7_), “normal” (GCG_8_), and “long” (≥GCG_9_ repeats). The distribution of *NIPA1* GCG repeat length in ALS and control samples is shown in Table 2. We found that 101 out of 590 *NIPA1* alleles in the ALS cohort harbored a 7 GCG repeat length (17.11%), as compared to 48 (14.4%) out of 334 control alleles (Figure 2A). A normal repeat length was found in 476 (80.7%) of the 590 ALS alleles and in 279 (85.5%) of the 334 control alleles (Figure 2A). Finally, a long RE was found in 13 (2.2%) of the 590 ALS alleles and in 5 (1.5%) of the 334 control samples (Table 2, Figure 2A). Therefore, *NIPA1* long alleles do not appear to be more frequent in ALS patients compared with controls (*p* = 0.392), although we can underline a statistically significant association (OR = 0.1) between REs’ borderline and the clinical phenotype of the disease. To verify the probable role of *NIPA1* long alleles as a modifier of disease phenotype, we investigated the associations of REs’ length and several clinical characters. Fisher’s test and χ^2^ did not show a difference between normal and short repeats with the age of onset, gender, and site of onset (Table 3). The correlation between gender and OR revealed a weak relationship between normal and short expansions (OR values fluctuate between 0.8 and 1.2) while a moderate association was shown with the long allele (OR = 3.375). Additionally, to evaluate the impact of RE length on the disease progression rate, we performed a Fisher’s exact test dichotomizing the ΔFS values in <0.7 and ≥0.7. The analysis results showed a statistically significant association between the long allele and ΔFS compared to the normal and short length (*p* = 0.02).

**Figure 3:**
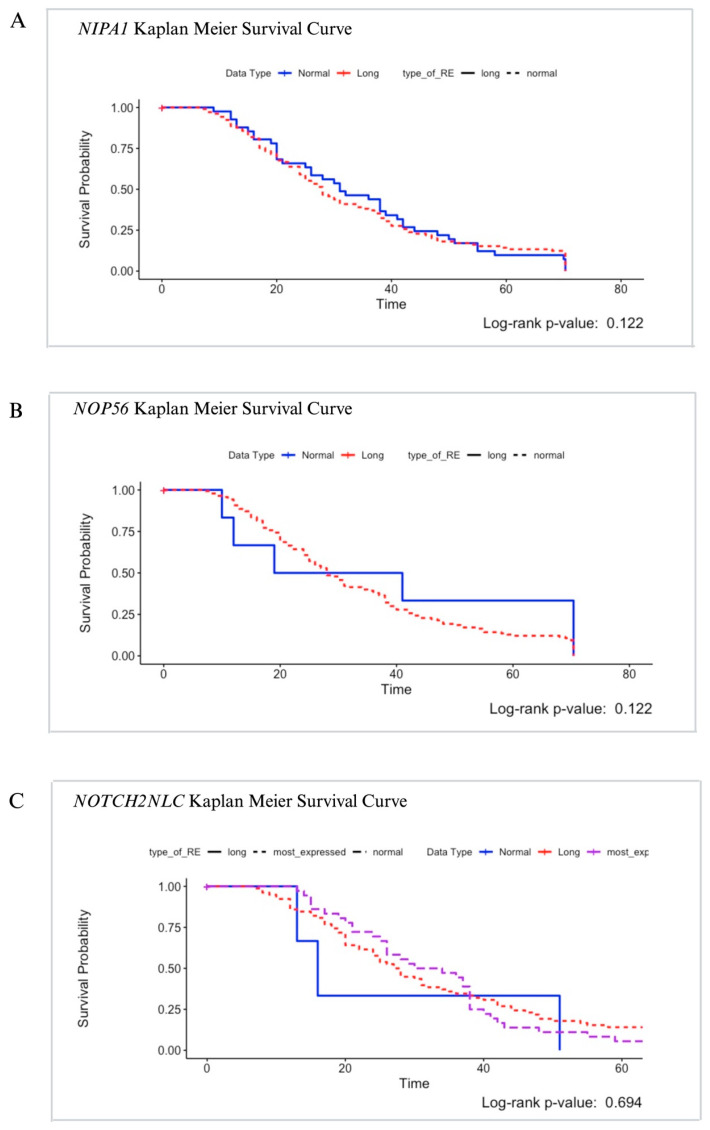
Kaplan–Meier survival curves of (**A**) *NIPA1*, (**B**) *NOP56,* and (**C**) *NOTCH2NLC* genes.

The estimated survival effect of patients’ GCG length and survival time was examined using Kaplan–Meier survival analysis. The Kaplan–Meier curve was generated to visualize the probability of survival over time based on the length of the repeat expansion. However, the log-rank test revealed that the observed differences in survival between groups did not reach statistical significance (*p* > 0.122). Consequently, we did not keep any significant association between the length of REs in *NIPA1* and survival time in our study cohort (Figure 3A).

In our cohort, 11 of 16 patients carrying the *C9orf72* pathological expansion showed the *NIPA1* normal allele (CCC_8_), while the other five showed a heterozygous pattern (GGC_7_-GGC_8_). Analysis using the Fisher test showed no evidence of an association between *C9orf72* carriers and the expansions in *NIPA1* (*p* = 0.105).

### 3.2. NOP56

An abnormal number of gene REs (>650) are correlated with the pathogenesis of SCA36, although this disease is considered a rare repeat expansion disorder. More than 60% of cases show phenotypic features of motoneuronal degeneration compatible with ALS [29]. For this reason, we wished to investigate the association between long repeats (GGCCTG_8_ and GGCCTG_9_) and ALS.

In our cohort, the sizes of REs in *NOP56* ranged from 3 to 9 repeats (GGCCTG_3_-GGCCTG_9_) in ALS patients and from 3 to 8 repeats (GGCCTG_3_-GGCCTG_8_) in controls subjects. We used the Cochran–Armitage test for trends to test whether the RE size of the alleles is associated with ALS. However, the RE distribution between ALS and control cases was similar (*p* = 0.40) with the most frequent alleles being GGCCTG_3_ (38.96%), GGCCTG_4_ (33.76%), and GGCCTG_6_ (67.96%) (Figure 2B).

The distribution of the *NOP56* GGCCTG repeat length in ALS and control samples is shown in Table 2. We found that 66 out of 590 *NOP56* alleles in the ALS cohort had a GGCCTG repeat length of 8 (11.1%), compared to 20 (5.98%) out of 334 control alleles. A repeat length of at least 9 (≥9) was found in 10 (1.7%) of the 590 ALS alleles and 3 (0.9%) of 334 control alleles (Table 2, Figure 2B).

Therefore, alleles with REs greater than 8 in the *NOP56* gene do not appear to be correlated with the disease (*p* > 0.99), so much so that the probability of finding this number of repeats is equal between cases and controls (OR = 0.93). In addition, we discovered no significant associations in the age of onset, gender, site of onset, family history, and ALSFRS-R scores between these two groups (*p* > 0.05) (Table 3).

As previously, we evaluated the impact of RE length by performing a Fisher’s exact test dichotomizing the ΔFS values into <0.7 and ≥0.7. Our findings revealed no significant association (*p* = 0.49) between long repeats (GGCCTG_8_ and GGCCTG_9_) and the prediction survival value.

The potential impact of patients’ GGCCTG repeat expansion length on survival time was assessed using Kaplan–Meier survival analysis. As before, upon conducting the log-rank test, we found that the differences in survival between the groups were not statistically significant (*p* > 0.122). Consequently, our analysis did not reveal any significant association between the length of GGCCTG REs in the *NOP56* gene and survival time within our study cohort (Figure 3B)

### 3.3. NOTCH2NLC

In this study, we used cut-off values for both pathogenic and normal sizes of *NOTCH2NLC* REs in accordance with previous work [21]: ≥60 repeats as pathogenic expansion, ≤43 as normal repeat size, and 44 to 59 as intermediate. The *NOTCH2NLC* GGC repeat size of the 295 ALS patients ranged from 7 to 15 (GGC_9_-GGC_15_), with 9 REs being the most prevalent size making up 66.8% of the total alleles.

The distribution of the *NOTCH2NLC* GGC repeat length in ALS and control samples is shown in Table 2. We found that 335 out of 590 *NOTCH2NLC* alleles in the ALS cohort had a GGC repeat length of 9 (56.8%), compared to 158 (47.3%) out of 334 control alleles. A repeat length greater than or equal to 15 was found in 7 (1.2%) of the 590 ALS alleles and in 2 (0.6%) of the 334 control alleles (Table 2, Figure 2C).

To obtain a statistical certainty of the data, we evaluated the association of the most common repeats (GGC_9_) and of the longest repeats (GGC ≥ 15) with several clinical characters. The statistical analysis showed no significant difference in the REs size of the longest repeats between ALS and control samples (*p* = 0.42). Furthermore, we discovered no significant associations in the age of onset, gender, site of onset, family history, and ALSFRS-R scores between the two groups analyzed (*p* = 0.33).

Examining the influence of patients’ GGC REs length on survival time involved employing the Kaplan–Meier survival analysis. After conducting the log-rank test, we established that the variations in survival between the groups did not achieve statistical significance (*p* > 0.694). Consequently, our investigation did not unveil any noteworthy correlation between the length of GGC repeat expansions in the *NOTCH2NLC* gene and survival time within the subjects under study (Figure 3C).

## 4. Discussion

ALS is a multifactorial heterogeneous disease in which long and/or intermediate repeats in several genes have been implicated. To the best of our knowledge, this is the first study conducted on ALS patients from southern Italy to investigate REs in three emerging disease-related genes. Despite previous studies suggesting a correlation between long allele expansions and ALS, our research in a cohort of ALS patients from southern Italy found no significant association between REs in *NIPA1*, *NOP56*, and *NOTCH2NLC* compared to the healthy controls. Furthermore, we did not observe any association between expanded motifs in these genes and different parameters such as gender, age at onset, site of onset, and ALSFRS-R scores.

*NIPA1* appears to be the third known enlarged genomic repeat motif linked to a higher risk for ALS, following *ATXN1* and *ATXN2*. It was first identified by Blauw and collaborators [15,16], and subsequently confirmed by Tazelaar et al. in 2019, that there is an association between long repeats in *NIPA1* and ALS risk. Furthermore, long REs have been shown to be positively associated with early disease onset and short survival [34,35]. In addition, several studies have been performed to find the possible role of *NIPA1* REs as modifiers of disease risk in *C9orf72* hexanucleotide repeat expansion carriers. However, the results do not boast a common conclusion: the study performed by Dekker et al. (2016) claimed to have found a high frequency of *NIPA1* REs in *C9orf72*-sALS cases, hypothesizing their role in modifying the *C9orf72* phenotype [36]. The study by Corrado et al. [37], consistent with the other two papers [34,38], concluded that the length of the *NIPA1* allele does not appear to affect the clinical features of *C9orf72* carriers.

Our study revealed that the frequency of *NIPA1* long alleles (≥GCG_9_ repeats) in ALS patients was not higher than that in healthy controls (4.4% vs. 3%). In addition, the results also confirmed no evidence of a relationship between *C9orf72* and NIPA1 in disease modulation (*p* = 0.105), consistent with the data reported by Corrado et al. in 2019. Interestingly, our study revealed an association between long repeats and disability progression and severity (ΔFS) (*p* < 0.02; Table 2). Despite the impossibility of drawing conclusions, it might be interesting to consider the potential implications of this result.

Consistent with findings in the Chinese [17], Japanese [39], and African [40] cohorts, our investigation within the South Italian cohort did not reveal any statistically significant association between *NOP56* allele frequencies and ALS cases. To the best of our knowledge, the frequency of REs in *NOP56* has never been described in European populations, and this is the first study on that. Despite the absence of a positive correlation, these shared outcomes contribute to the global comprehension of *NOP56’s* role in ALS, emphasizing the importance of cross-cultural studies in unraveling the genetic complexities of this neurodegenerative disease. *NOP56* is a causative gene of a specific SCA subtype, SCA36. It is also known that some patients with SCA36 clinically exhibit an ALS phenotype. Both diseases cause progressive late-onset motor neuron degeneration potentially caused by the expansion of hexanucleotide portions: *NOP56* in the case of SCA36 [39] and *C9orf72* in the case of ALS [7]. The motor neuron portion involved in these two diseases is different, but some patients are carriers of pathogenic repeats of GGCCTG in *NOP56* [18], suggesting an overlap of genetic and phenotypic events between ALS and SCA36 [29,41]. To date, there have been few studies on the association of repeat expansion and *NOP56* with ALS, and further research into the typical role of this gene will be needed to shed light on how it contributes to the disease.

In the present study, none of the ALS patients examined exhibited the GGC repeat expansion in the *NOTCH2NLC* gene. While investigations into the association between these REs and ALS remain limited, noteworthy insights have been gleaned from related studies. Notably, a Japanese study by Yuan et al. (2020) reported the identification of four ALS patients (the studied population included 545 ALS patients and 1305 healthy controls) with anomalous GGC repeat expansions, suggesting a potential modifying factor for ALS in the context of *NOTCH2NLC* [21]. Furthermore, ALS patients with abnormal GGC-repeat expansion tended to exhibit a severe phenotype and rapidly progressing disease, with a relatively small number of GGC repeats (mean: 84, Range: 44–143) [24]. Beyond the extremes of repeat lengths, the study also sheds light on the significance of intermediate repeats (42 to 47), as identified in patients with leukoencephalopathy and controls with corticobasal syndrome and progressive supranuclear palsy. While the details and frequencies of these intermediate repeats remain undisclosed, their absence in healthy controls hints at a potential association with neurodegenerative diseases, including ALS and dementia [24,42,43]. More recently, Manini and collaborators investigated an Italian cohort of ALS patients reporting the absence of pathological expansion [22]. In line with this, our study confirms this finding in our samples. It also shows that there is no association between different repeat lengths in both ALS and control populations.

There are some limitations to our study that need to be considered: (i) the control group included both healthy samples (*n* = 31) and individuals diagnosed with various neurological disorders (*n* = 136). Although this group is clinically and genetically well-differentiated compared to the ALS population, the use of a cohort of healthy controls could eliminate potential confounding factors that may influence the interpretation of the results; (ii) the relatively small sample size in some of the subgroups may limit the statistical power to detect significant associations (e.g., the small number of *C9orf72* ALS patients in the three repeat expansion groups).

Despite these limitations, on the basis that the replication of genetic associations in populations can provide support for the disease causation scenario and, if not replicated, can also raise new questions about undelight pathogenetic processes, our present study provides valuable insights on the frequency of REs in *NIPA1*, *NOP56*, and *NOTCH2NLC* in a cohort of South Italian ALS cases and highlights the need for larger and more comprehensive studies to validate and extend our findings.

## 5. Conclusions

This is the first study to screen ALS patients from southern Italy for REs in *NIPA1*, *NOP56*, and *NOTCH2NLC* genes Our results did not conclusively support the hypothesized role of repeat lengths in *NIPA1*, *NOP56*, and *NOTCH2NLC* as determinants of ALS risk. Furthermore, the multiple statistical analyses scrutinizing potential associations between clinical parameters and repeat length did not yield discernible correlations.

Efforts to discover novel ALS disease-modifying factors are a critical goal to promote the development of personalized strategies for diagnosis, prognosis, and therapy. In particular, studies on the association of intermediate repeat expansions with the clinical features of ALS or disease progression could pave the way for refining individual prognostic predictions and improving ALS clinical trial design, as is already happening with *ATXN2.*

## Figures and Tables

**Table 1:** Demographic and clinical characteristics of the South Italian ALS cohort.

South Italian ALS Cohort (*n* = 302)	
Age at onset, years, mean (± SD)	61.6 (± 12.6)
Male, n (%)	163 (54%)
Sporadic ALS, n (%)	275 (91%)
Familiar ALS, n (%)	27 (9%)
*C9orf72*-ALS, n (%)	16 (5.3%)
*FUS*-ALS, n (%)	2 (0.7%)
*SOD1*-ALS, n (%)	1 (0.3%)
*TARDBP-ALS*	6 (2%)
Family history of NDs, n (%)	
Yes	68 (22.5%)
No	234 (77.5%)
Region of symptom onset, n (%)	
Spinal	209 (69.2%)
Bulbar	61 (20.2%)

Family history of NDs includes: Alzheimer’s disease (AD), Parkinson’s disease (PD, multiple sclerosis (MS), and dementia.: Data not available for region of symptoms onset in *n* = 32 (10.6%).

**Table 2:** Frequencies of *NIPA1*, *NOP56,* and *NOTCH2NLC* repeat alleles in ALS and control samples.

Repeat Alleles	ALS	Controls	*p*-Value	Odd Ratio
***NIPA1***	*n* (%)	*n* (%)		
Short (7 repeats)	101 (17.1%)	48 (14.4%)	0.39	0.1
Normal (8 repeats)	476 (80.7%)	279 (85.5%)		
Long (≥9 repeats)	13 (2.2%)	5 (1.5%)		
***NOP56***				
8 repeats	66 (11.1%)	20 (5.98%)	0.40	0.93
≥9 repeats	10 (1.7%)	3 (0.9%)		
***NOTCH2NLC***				
9 repeats	335 (56.8%)	158 (47.3%)	0.42	0.8
≥15 repeats	7 (1.2%)	2 (0.6%)		

**Table 3:** Association analysis between *NIPA1*, *NOP56,* and *NOTCH2NLC* allele lengths and age at onset and gender in ALS and control samples.

***NIPA1***						
**GCG repeat** **allele**	Cohort	Age of onset (mean *)	*p*-value (Paired *t*-test)	Gender	*p*-value (Fisher’s exact test)	OR (Batista Pike)
**Short (7)**	ALS	61.74 (13.9)	0.2544	43 M 47 F	0.7137	0.835
	CTRLs	63.07 (16.8)		23 M 21 F		
**Normal (8)**	ALS	61.45 (12.1)		149 M 128 F	0.4251	1.192
	CTRLs	63.07 (16.8)		78 M 79 F		
**Long (≥9)**	ALS	58.8 (11.7)		9 M 4 F	0.2545	3.375
	CTRLs	57.9 (15.1)		2 M 3 F		
***NOP56***						
**GGCCTG repeat allele**	Cohort	Age of onset (mean *)	*p*-value (Paired *t*-test)	Gender	*p*-value (Fisher’s exact test)	OR (Batista Pike)
**8 REs**	ALS	65.08 (10.1)	0.6837	32 M 30 F	0.2118	1.981
	CTRLs	63.13 (16.7)		7 M 13 F		
**≥9 REs**	ALS	61.27 (13.4)		4 M 6 F	0.5594	0.3333
	CTRLs	62.86 (16.3)		2 M 1 F		
***NOTCH2NLC***						
**GGC repeat allele**	Cohort	Age of onset (mean *)	*p*-value (Paired *t*-test)	Gender	*p*-value (Fisher’s exact test)	OR (Batista Pike)
**9 REs**	ALS	61.66 (12.7)	0.3634	95 M 101 F	0.4084	0.8
	CTRLs	62.9 (16.6)		61 M 52 F		
**≥15 REs**	ALS	61.05 (13.9)		4 M 3 F	0.5	0
	CTRLs	72.8 (12.5)		2 M 0 F		

* Mean (±standard deviation) values are displayed. OR= odd ratio. CTRLs = control cases.

## Data Availability

The data presented in this study are available in results and Supplementary Material sections.

## Supplementary Materials

The following supporting information can be downloaded at: https://www.mdpi.com/article/10.3390/cells13080677/s1, Figure S1: Results of conventional polymerase chain reaction (PCR) and repeat PCR (RP-PCR), Table S1: Set of primers used in fragment-length analysis and repeat-primed -PCR for NIPA1, NOP56 and NOTCH2NLC genes study.
