# Supplementary material for: Investigating Repeat Expansions in NIPA1, NOP56, and NOTCH2NLC Genes: A Closer Look at Amyotrophic Lateral Sclerosis Patients from Southern Italy
Source: Cells. 2024 Apr 14;13(8):677. doi: 10.3390/cells13080677 (PMC11049433; doi:10.3390/cells13080677)

Supplementary Table S1. Set of primers and conditions used in fragment-length analysis and repeat-primed -PCR for *NIPA1, NOP56* and *NOTCH2NLC* genes study.

Gene	Experiment	Primer name	Sequences
NIPA1	fragment-length analysis	Forward	5'-[6FAM]CGGAATGGGGACTGCAGCT-3'
		Reverse	5′-ACGATGCCCTTCTTCTGTAGCA-3'
NOP56	fragment-length analysis	Forward	5'-[6FAM]TTTCGGCCTGCGTTCGGG-3'
		Reverse	5'-AACGCAACCTCAGCGTCT-3'
	repeat-primed PCR	RE primer	5'-TACGCATCCCGTTTGAGACGCAGGCCCAGGCCCAGGCCCAGGCC-3'
		Universal Primer	5'-TACGCATCCCGTTTGAGACG-3'
NOTCH2NLC	fragment-length analysis	Forward	5'-[VIC]CATTTGCGCCTGTGCTTCGGAC-3'
		Reverse	5'- AGAGCGGCGCAGGGCGGGCATCTT-3'
	repeat-primed PCR	RE primer	5'-[6FAM]GGCATTTGCGCCTGTGCTTCGGACCGT-3'
		M13- (GGC)4 (GGA)2-R	5'-CAGGAAACAGCTATGACCTCCTCCGCCGCCGCCGCC-3'
		M13- linker R	5'-CAGGAAACAGCTATGACC-3'

For *NIPA1*, the amplicon was PCR amplified using the same protocols described by Blauw et al., 2012 [16]. Specifically, the reaction mixture was the following: 0.4 µl long-range Taq polymerase (Long PCR Enzyme Mix), 200 nM dNTPs, 100 nM forward and reverse primer, 5% dimethylsulfoxide, 1 µl PCR buffer with MgCl2 (Long PCR Enzyme Mix) and 50 ng of genomic DNA. The PCR reaction conditions were as follows: initial denaturation for 4 minutes at 95°C, followed by 35 cycles at 94°C for 20 seconds, 55°C for 30 seconds, 68°C for 4 minutes and a final extension at 68°C for 10 minutes.

For the genotyping of *NOP56* and *NOTCH2NLC*, we used a two-step strategy. Briefly, the first step was the fluorescent length fragment genotyping to obtain the number of repeats of the wild allele. In the case of homozygotes (Figure S1-A-B), the second step was performing the repeat primer polymerase reaction to determine the presence of the expansion (Figure S1-A-B). Classically, pathological expansions were defined by the characteristic saw-tooth pattern visualization (Figure S1-C).

For *NOP56* primed PCR we followed the protocol already described by [29]. The final 25 µl of reactions containing the following components: 60 ng of DNA, 200 µM of each dNTP, 1.5 mM MgCl2, 5 µl of 360 GC Enhancer (Applied Bio-systems), 1.25 U AmpliTaq Gold4® 360 DNA Polymerase, 1x buffer (Applied Biosys-tems), 0.8 µM reverse universal primer, 0.8 µM forward primer, and 0.08 µM re-peat-specific primer. The reaction mixture was denatured at 95°C for 10 minutes and subjected to 40 cycles (95°C for 1 minute, 63.5°C for 1 minute, 72°C for 4 minutes, with an additional 10 seconds added each cycle). The mixture was held at 72°C for 10 minutes for the final extension [39].

For *NOTCH2NLC*, the repeat primed PCR mix was made following the protocol described by [23]. The mixute containing the following components: 0.25 U of PrimeSTAR GXL DNA Polymerase, 1x of PrimeSTAR GXL Buffer, 200 µM each of dATP, dTTP, dCTP (Takara Bio), and 7-Deaza-2′-deoxy-guanosine-5′-triphosphate (Sigma-Aldrich), 5% dimethyl sulfoxide (Sigma-Aldrich), 1 M betaine (Sigma-Aldrich), 0.3 µM each of primer mix, and 100 ng of genomic DNA in a total reaction volume of 10 µl. After incubation at 98 °C for 10 min, the cycling conditions were set as follows: 16 cycles of 98 °C for 30 s, 66 °C

for 1 min with a reduction of 0.5 °C per cycle, and 68 °C for 8 min, followed by 29 cycles of 98 °C for 30 s, 58 °C for 1 min, and 68 °C for 8 min. Finally, a final elongation step of 68 °C for 10 min was performed. The ramp rate of all cycling steps was adjusted to 0.5 °C per second.

Figure. S1. Results of conventional polymerase chain reaction (PCR) and repeat PCR (RP-PCR). The x-axis shows the product size in bp, and the y-axis shows the peak height measured from the signal intensity of the fluorescently labeled PCR products. Panel (A) illustration of *NOP56*: the first panel illustrates the presence of two normal-sized *NOP56* alleles (106bp/118bp; 4/6 repeats); the second panel presents the identification of homozygotes detected by conventional PCR; the third panel represents the RP-PCR results. Panel (B) illustration of *NOTCH2NLC*: the first panel demonstrates the presence of two normal-sized *NOTCH2NLC* alleles (185bp/193bp; 9/12 repeats); the second panel identifies homozygotes detected by conventional PCR; the third panel depicts the RP-PCR results. Panel (C): Typical saw-tooth pattern of an expanded allele.

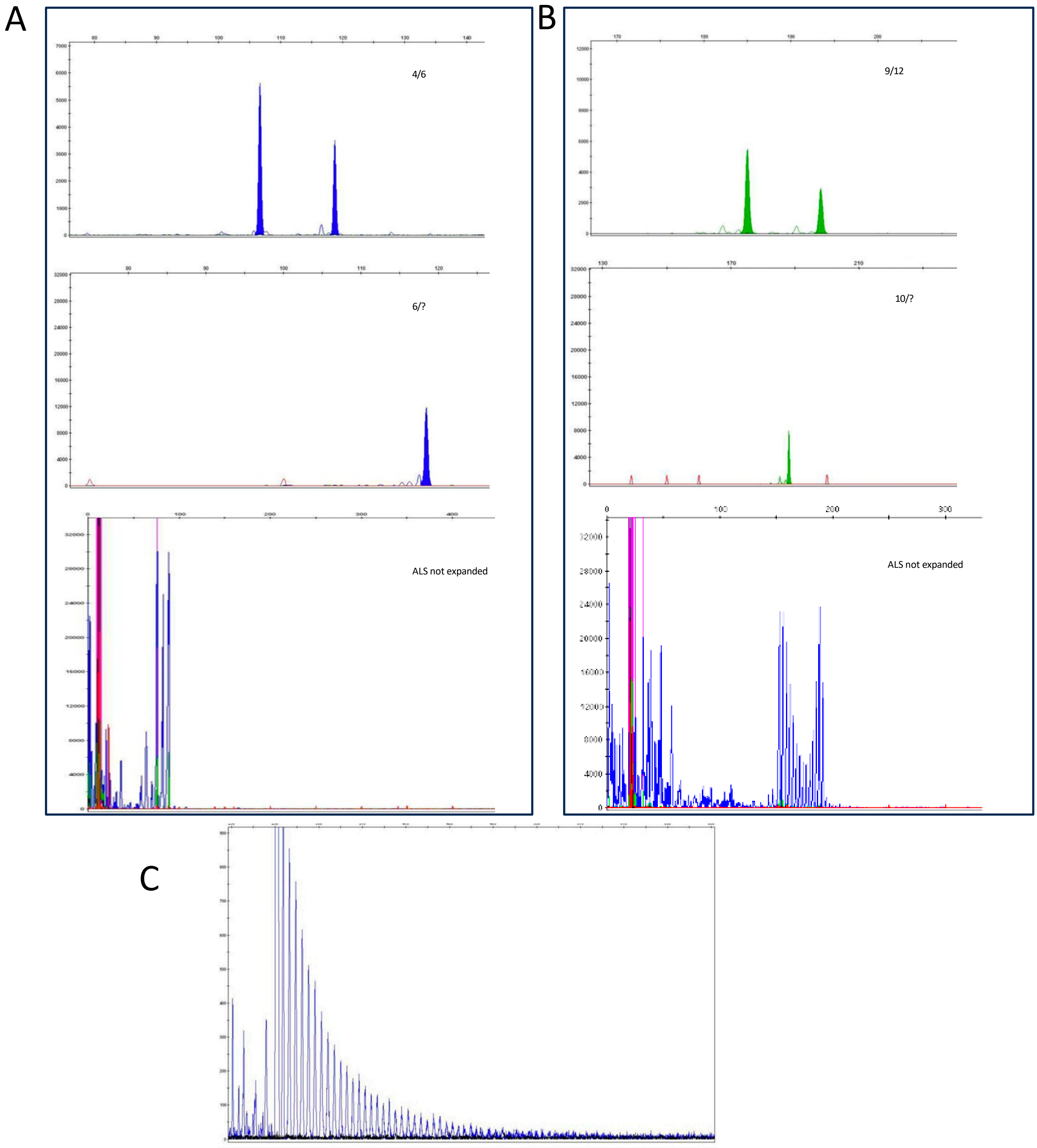

Supplement: Supplementary file 1 [file cells-13-00677-s001.zip › cells-2899262-supplementary.pdf]
